# Early Myasthenia Gravis Activities of Daily Living (MG-ADL) Response to Ravulizumab in Acetylcholine Receptor-Positive Generalized Myasthenia Gravis Refractory: A Case Report From Latin America

**DOI:** 10.7759/cureus.86874

**Published:** 2025-06-27

**Authors:** Manuel Orellana, Gabriel Bustamante

**Affiliations:** 1 Neurology, Hospital Dr. Franco Ravera Zunino, Rancagua, CHL; 2 Neurology, Pontificia Universidad Católica de Chile, Santiago, CHL; 3 Neurology, Hospital de la Dirección Previsional de Carabineros de Chile, Santiago, CHL

**Keywords:** acetylcholine receptor antibody, clinical case report, complement inhibitor, myasthenia gravis, ravulizumab

## Abstract

This case report details the first use of ravulizumab in Chile for a patient with acetylcholine receptor (AChR)-positive myasthenia gravis (MG) who did not respond to rituximab (RTX). A 26-year-old female presented with progressive MG symptoms, including fatigue and bilateral ptosis. Despite conventional treatment and thymectomy, she experienced multiple myasthenic crises. Following the lack of response to RTX, ravulizumab was initiated, resulting in a significant improvement in the Myasthenia Gravis Activities of Daily Living (MG-ADL) score in less than two weeks. Ravulizumab, a monoclonal antibody that inhibits complement component C5, was approved for MG in 2022 and is being assessed for efficacy in neurological disorders. This report highlights its potential as a therapy for MG in the absence of robust local evidence, offering a novel therapeutic option for managing this debilitating condition.

## Introduction

Myasthenia gravis (MG) is a rare autoimmune disorder characterized by muscle weakness due to autoantibody-mediated and complement-involved mechanisms, affecting the neuromuscular junction [1]. Most individuals with MG possess pathogenic autoantibodies targeting the extracellular portion of acetylcholine receptors (AChR), often linked to an abnormal thymus [2]. Approximately 85% of MG patients have autoantibodies against the muscle AChR (AChR-MG), while about 5% possess autoantibodies against muscle-specific kinase (MuSK-MG). In the remaining 10% of cases, no autoantibodies are detected using classical diagnostics for AChR and MuSK antibodies, classifying them as seronegative MG (SN-MG) [3,4].

The management of MG has primarily depended on acetylcholinesterase inhibitors (AChE-I), corticosteroids, and immunosuppressants to alleviate symptoms and control the autoimmune response [5]. Approximately 10-15% of people with MG fail to respond to conventional immunotherapy and are considered to have refractory disease [6,7].

Ravulizumab is a humanized monoclonal antibody targeting complement component C5, administered at dosing intervals of eight weeks [8]. It has been approved for the treatment of paroxysmal nocturnal hemoglobinuria [9] and atypical hemolytic uremic syndrome [10]. For neurological indications, ravulizumab has been approved for patients with neuromyelitis optica spectrum disorder (NMOSD) and AChR-positive MG in 2022. Currently, it is being investigated in pediatric patients with NMOSD and generalized MG (gMG) in clinical trials NCT05346354 and NCT05644561, respectively, both of which are recruiting. Additionally, study NCT06291376 is planned to evaluate the use of ravulizumab in proliferative IgA nephropathy, while study NCT04564339 on proliferative lupus nephritis or IgA nephropathy is active but not yet recruiting.

For the MG trial, the trial met the primary end point reduction of Myasthenia Gravis-Activities of Daily Living Profile (MG-ADL) (ravulizumab: −3.1, placebo: −1.4, treatment difference: −1.6, p = 0.001) at 23 weeks. In addition, the proportion of patients experiencing an improvement of the Quantitative Myasthenia Gravis (QMG) total score of at least five points was higher in the ravulizumab group (30.0 versus 11.3%) at 23 weeks [11]. Participants who completed the randomized controlled period (RCP) could receive ravulizumab in the open-label extension. Patients who switched from placebo in the RCP to ravulizumab in the OLE showed rapid improvements in the MG-ADL score, which were maintained through 138 weeks (least-squares mean change from OLE baseline at week 164: -2.1 (95% CI: -3.3, -0.9); p<0.0005). QMG total score improvements were also maintained in patients continuing ravulizumab in the open-label extension (OLE), and the scores improved from OLE baseline in patients switching from placebo to ravulizumab. Ravulizumab was well tolerated; no meningococcal infections were reported [12,13].

The use of ravulizumab in Chile for patients with generalized AChR-positive MG is supported by very limited evidence. This report presents the first case and reviews the existing literature.

## Case presentation

A 26-year-old female patient without a morbid history consulted in February 2021 for a six-month progressive course of asymmetric bilateral blepharoptosis greater to the right, binocular diplopia, fatigue, and dyspnea progressively increasing during the day.

**Figure 1:**
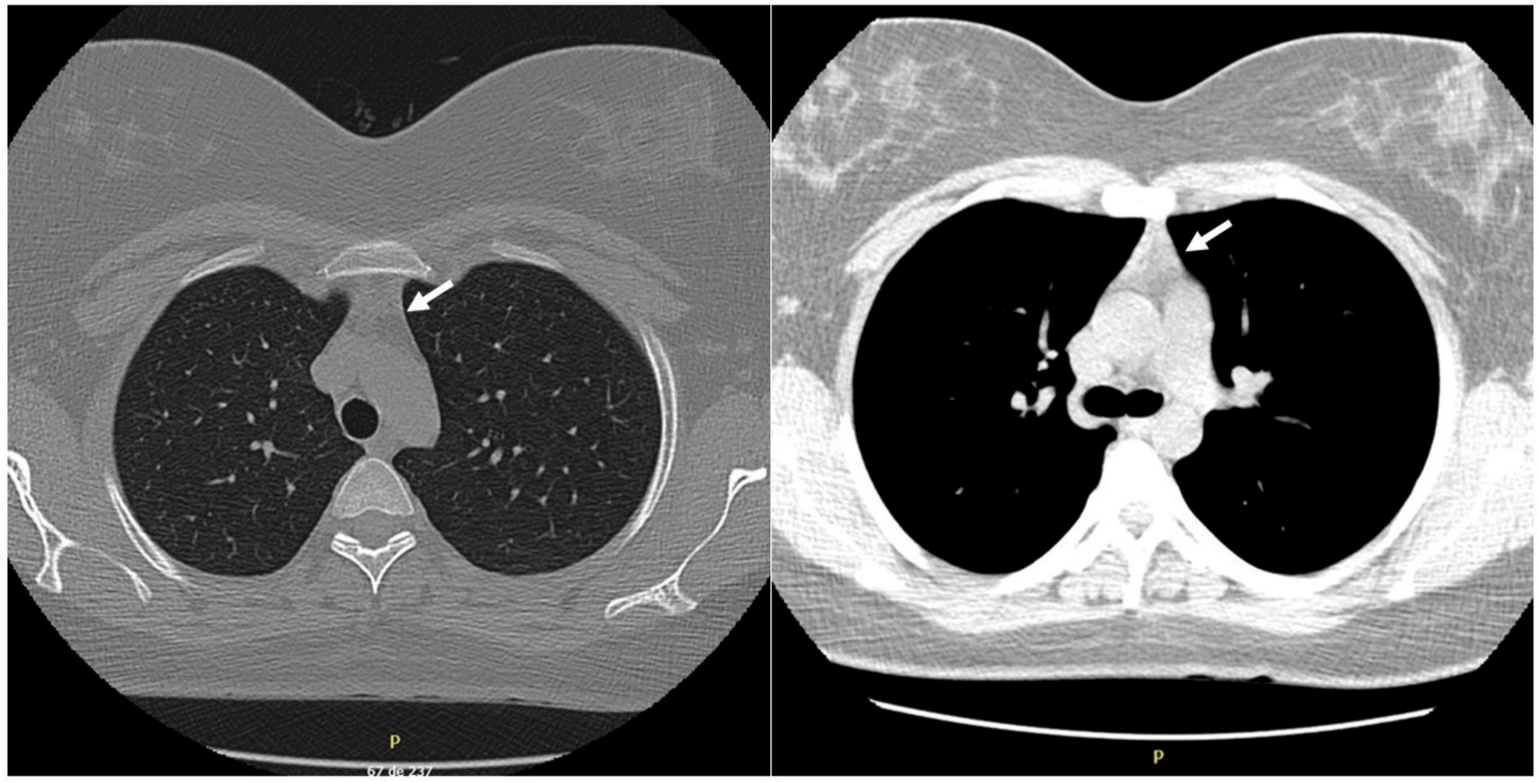
Axial non-contrast chest computed tomography (CT) scans. The left image, obtained in the bronchial phase, shows normal lung parenchyma and mediastinal anatomy without evidence of masses (white arrow). The right image, acquired in the mediastinal phase, demonstrates residual thymic tissue in the anterior mediastinum (white arrow), with no signs of thymoma. These findings are consistent with persistent thymic remnants in a patient with acetylcholine receptor antibody-positive generalized myasthenia gravis.

The diagnosis of myasthenia gravis was considered, and a study was carried out by means of a 3 Hz repetitive stimulation test. For this purpose, the abductor digiti minimi and right nasalis muscles were used as effectors, stimulating the ipsilateral ulnar and facial nerves. The result showed a significant drop (greater than 10%) in amplitude. In addition, antibodies against AChR (anti-AChR) and anti-MuSK were tested using radioimmunoassay (RIA) methodology in the laboratories of the Mayo Clinic. Serological results indicated positivity for anti-AChR (9.55 nmol/L, reference value: < 0.02 nmol/L) and negativity for anti-MuSK antibodies (0.00 nmol/L, reference value: 0.00-0.002 nmol/L). The study was complemented with a computed tomography (CT) of the thorax without contrast, which showed the presence of scarce thymic tissue in the anterior mediastinum, without masses suggestive of thymoma (Figure 1). With these results, the diagnosis of generalized myasthenia gravis seropositive for anti-AChR antibodies was established, with thymic tissue present.

The patient started pharmacological treatment with oral pyridostigmine at 90 mg per day, along with prednisone 60 mg daily and azathioprine (AZA) 150 mg daily. The pyridostigmine dose was progressively increased during the first year to 360 mg/day (six tablets). During periods of symptom exacerbation, it was further increased to 480 mg/day (eight tablets), a dose that was maintained for an extended period due to persistent clinical activity. Additionally, the patient underwent a thymectomy in October 2022. Despite these treatments, she experienced eight myasthenic crises by December 2024, all requiring management in the intensive care unit with plasmapheresis (PLEX) or intravenous immunoglobulin (IVIg). As a complication of prolonged corticosteroid use, the patient developed morbid obesity, leading to bariatric surgery in September 2024.

Due to the refractory nature of the clinical picture, the decision was made to change the immunosuppressive treatment to rituximab (RTX) in July 2023, completing a total of 10 infusions, with a four-week induction regimen based on body surface area, followed by 1,000 mg intravenous every six months. Despite maintaining the immunosuppressive regimen at maximum doses, together with pyridostigmine at 60 mg (five tablets daily orally) and prednisone at 10 mg daily orally, the patient experienced a decompensation four months post infusion of RTX. This decompensation was presented with invalidating fatigability, diplopia, and blepharoptosis, with a score on the MG-ADL of 10. The MG-ADL scale is a validated eight-item, patient-reported outcome measure developed to quantify disease severity and functional impairment in MG [14]. Each item is scored from 0 (normal) to 3 (severe), resulting in a total score ranging from 0 to 24. Higher scores indicate greater disability and symptom burden. A change of ≥2 points in the MG-ADL score is generally considered the minimal clinically important difference (MCID) [15], indicating a meaningful improvement in the patient’s condition. Due to the persistent refractoriness of symptoms, ravulizumab was initiated in January 2025, starting with a loading dose of 2,700 mg, followed by maintenance doses of 3,300 mg on day 15 and every eight weeks thereafter. Prior to initiating ravulizumab, the patient was vaccinated against ACWY and B serogroups *Neisseria meningitidis* and *Streptococcus pneumoniae*.

**Figure 2:**
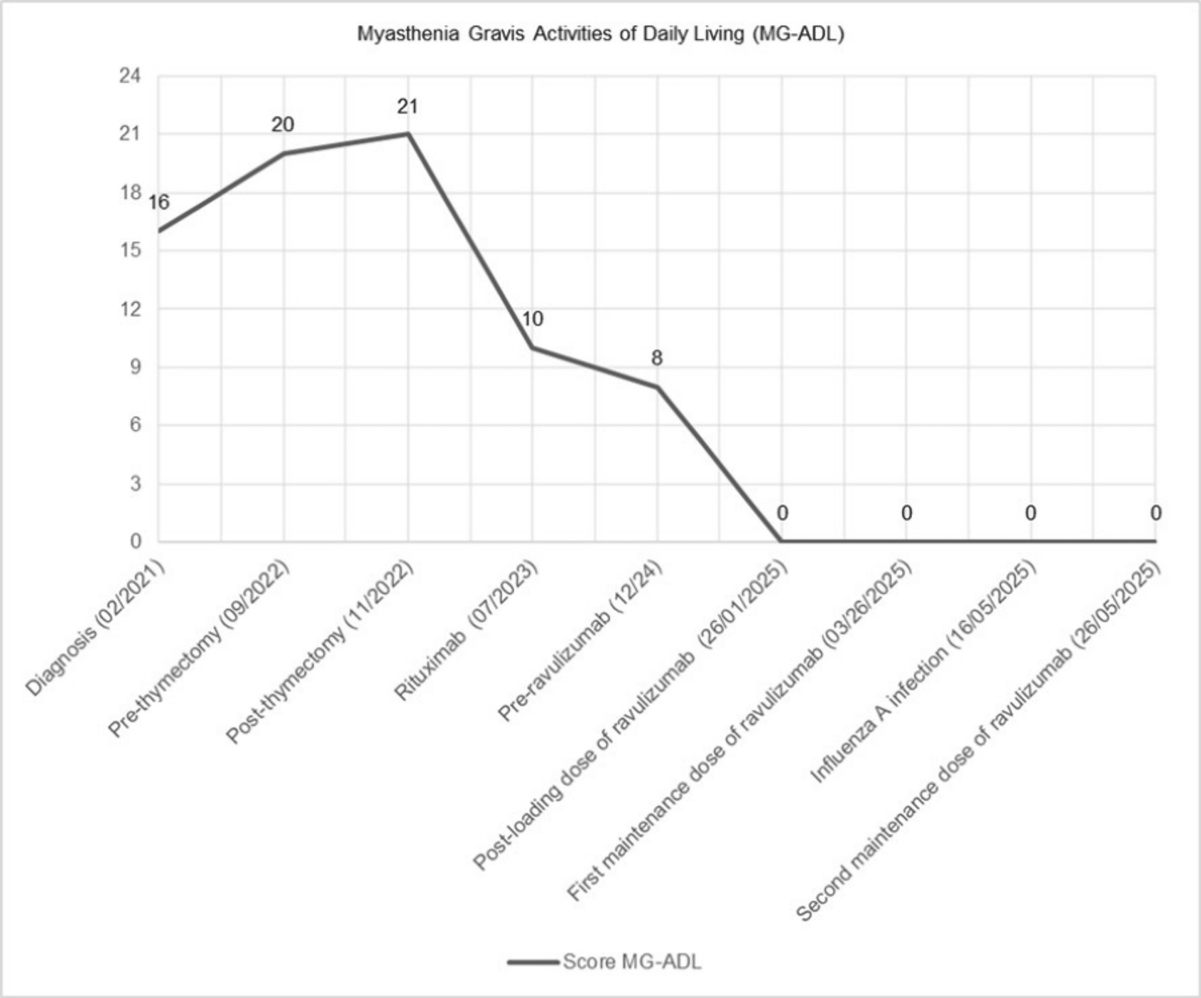
MG-ADL score evolution in the patient treated with ravulizumab. The patient's score increased after diagnosis and peaked post thymectomy. Following rituximab infusion (07/2023), the MG-ADL score improved markedly. Subsequent administration of ravulizumab (starting with the loading dose on 01/26/2025) led to complete resolution of symptoms (score = 0), which was sustained through maintenance dosing and during an intercurrent Influenza A infection. This trajectory highlights the potential of ravulizumab to achieve and maintain clinical remission in refractory MG.

During follow-up, the patient exhibited a marked reduction in the MG-ADL score, reaching a value of 0 two weeks after the loading dose of ravulizumab (Figure 2). No further clinical deterioration has been observed to date, despite the occurrence of an influenza A infection.

The patient has discontinued AZA and prednisone, and the dose of pyridostigmine has been reduced from 480 mg/day to only 180 mg/day.

## Discussion

Traditional treatments such as non-steroidal immunosuppressive therapies have slow effects and problematic side effects. Habit et al. established that the median time to first response was 2.1 weeks for a ≥2-point reduction in MG-ADL scores and 4.1 weeks for a ≥3-point reduction in QMG scores [16]. These findings suggest that many patients experience significant symptom improvement within a few weeks of starting ravulizumab. In our case, the patient showed clinical improvement in less than two weeks, highlighting the rapid onset of action of the molecule and its ability to prevent damage at the neuromuscular junction.

The use of RTX, a chimeric monoclonal antibody against CD20 that depletes circulating B cells, has emerged as an option in refractory cases. However, the efficacy of RTX in refractory AChR-Ab+ MG is uncertain [5]. Moreover, in patients with refractory MG AChR+, RTX, it generally requires several weeks to achieve its full therapeutic effect. However, in certain cases, clinical improvement is not sustained, highlighting the need to explore innovative therapeutic alternatives.

In this context, ravulizumab has proven beneficial, even in patients already undergoing RTX treatment. Konen et al. reported the first case where ravulizumab achieved significant neuromuscular function improvement within just one week, with these benefits maintained over a 19-week follow-up in a patient experiencing a myasthenic crisis [17]. This finding underscores the potential of complement inhibitors as an effective strategy to overcome refractoriness in such crises.

Treatment with ravulizumab has led to a reduction in pyridostigmine from 480 to only 180 mg daily and the discontinuation of azathioprine and prednisone. This is beneficial by reducing the cumulative dose of corticosteroids and the adverse effects associated with these drugs, such as obesity, Cushing's syndrome, osteoporosis, avascular necrosis of the femoral head, metabolic syndrome, acne, and peptic ulcer.

It is noteworthy that the patient had an influenza respiratory infection and, despite being treated with ravulizumab, did not experience complications related to the infection or exacerbation of MG symptoms.

It is currently necessary to expand the safety study of ravulizumab during pregnancy to ensure appropriate family planning, as it is likely that, given the positive results, young patients of childbearing age will consider this need.

## Conclusions

In conclusion, this case report demonstrates ravulizumab’s potential as a rapid and effective therapeutic option for AChR+ MG when conventional treatments fail. By inhibiting complement component C5, ravulizumab offers a novel mechanism of action that has contributed to its success in this case. Additionally, the use of this monoclonal antibody reduced the required dosage of corticosteroids and eliminated the need for immunosuppressants, thereby decreasing the occurrence of adverse events. This report supports the exploration of ravulizumab as a viable therapy in similar clinical contexts, despite the limited local evidence.
